# Evaluation of oral health-related quality of life among Sudanese schoolchildren using Child-OIDP inventory

**DOI:** 10.1186/1477-7525-8-152

**Published:** 2010-12-23

**Authors:** Nazik M Nurelhuda, Mutaz F Ahmed, Tordis A Trovik, Anne N Åstrøm

**Affiliations:** 1 Department of Clinical Dentistry, Faculty of Medicine and Dentistry - University of Bergen, Bergen, Norway; 2 Centre for International Health, Faculty of Medicine and Dentistry - University of Bergen, Bergen, Norway; 3 Liverpool University Dental Hospital, UK; 4 Department of Clinical Dentistry - Preventive Dental Care, Faculty of Medicine and Dentistry, University of Bergen, Bergen, Norway

## Abstract

**Background:**

Information on oral health-related quality of life, in addition to clinical measures, is essential for healthcare policy makers to promote oral health resources and address oral health needs.

**Objectives:**

This paper aimed at evaluating the psychometric properties of the Arabic version of Child-OIDP, estimating the prevalence, severity and causes of oral impacts on daily performances in 12-year-old public and private school attendees in Khartoum State and to identify socio-demographic and clinical correlates of oral impacts as assessed by the Child-OIDP inventory.

**Methods:**

The Child-OIDP questionnaire was translated into Arabic was administered to a representative sample of 1109 schoolchildren in Khartoum state. Clinical measures employed in this study included DMFT index, Gingival index, Plaque index and Dean's index. A food frequency questionnaire was used to study the sugar-sweetened snack consumption.

**Results:**

The instrument showed acceptable psychometric properties and is considered as a valid, reliable (Cronbach's alpha 0.73) and practical inventory for use in this population. An impact was reported by 54.6% of the schoolchildren. The highest impact was reported on eating (35.5%) followed by cleaning (28.3%) and the lowest impacts were on speaking (8.6%) and social contact (8.7%). Problems which contributed to all eight impacts were toothache, sensitive teeth, exfoliating teeth, swollen gums and bad breath. Toothache was the most frequently associated cause of almost all impacts in both private and public school attendees. After adjusting for confounders in the 3 multiple variable regression models (whole sample, public and private school attendees), active caries maintained a significant association with the whole sample (OR 2.0 95% CI 1.4-2.6) and public school attendees (OR 3.5 95% CI 2.1-5.6), and higher SES was associated with only public school attendees' Child-OIDP (OR 1.9 95% 1.1-3.1).

**Conclusion:**

This study showed that the Arabic version of the Child-OIDP was applicable for use among schoolchildren in Khartoum. Despite the low prevalence of the dental caries pathology (24%), a significant relationship, with an average moderate intensity was found with OHRQoL. Focus in this population should be on oral health education, improving knowledge of the prospective treatment opportunities and provision of such services.

## Introduction

Health is defined as the complete physical, mental and social well-being and not merely the absence of disease or infirmity. This health triangle is a key concept in achieving acceptable general and oral health-related quality of life (OHRQoL) [1]. The majority of studies on evaluation of oral health status was carried out using clinical measures only, however, OHRQoL instruments should be used in conjunction with them [2]. The perceived OHRQoL may vary between cultures, therefore, the psychometric properties of OHRQoL inventories should be assessed whenever applied in new socio-cultural contexts [3].

In literature a number of OHRQoL measures have been developed to assess and describe the oral impacts on people's quality of life. Five of these instruments were designed to assess the OHRQoL in children specifically. These include the following questionnaires: Child Perception Questionnaire (CPQ _11-14_), the Michigan OHRQoL scale, the Child Oral Health Impact Profile (Child-OHIP), the Early Childhood Oral Health Impact Scale (ECOHIS) and the Child Oral Impact on Daily Performance (Child-OIDP). In line with the WHO's International Classification of impairments, disabilities and handicaps [4], the Child-OIDP focuses on measuring the ultimate impacts of disabilities and handicaps thus capturing more proximal and intermediate impacts such as pain, discomfort, functional limitation and dissatisfaction with appearance. This inventory, applied in the present study, has the ability to provide information on condition specific impacts whereby the respondent attributes the impacts to specific oral conditions or diseases; thus contributing to the needs assessment and the planning of oral health care services [5]. The Child-OIDP was initially developed (in English) in Thailand [6] and has shown to be valid and reliable when applied to children in the United Kingdom [7], France [8], Tanzania [9], Peru [10], Brazil [11], Spain [12] and Italy [13].

The present study is part of a school-based survey conducted in Khartoum state, Sudan [14]. The results of this survey revealed that the mean DMFT of 12-year-old schoolchildren was 0.4 and that almost one quarter (24%) of these children had caries experience (DMFT > 0). Despite the low prevalence and severity of caries, almost three quarters (73.8%) of the schoolchildren were dissatisfied with their oral health. The caries experience was found to be associated with high socioeconomic status [14] and high levels of *Streptococcus sobrinus *in saliva [15].

Information on the OHRQoL of this population should add to the knowledge on dental caries by determining the magnitude of impact of poor dentition status on children's everyday activities. Reported impacts may put more emphasis on developing oral health promotion and care programmes.

This paper aimed at evaluating the psychometric properties of the Arabic version of Child-OIDP and to estimate the prevalence, severity and causes of oral impacts on daily performances in 12-year-old public and private school attendees in Khartoum State. Secondly, this study set out to identify socio-demographic- and clinical correlates of oral impacts as assessed by the Child-OIDP inventory.

## Materials and methods

### Sampling procedure

Khartoum state is divided into 7 main localities (*Khartoum, Jabal Awliya, Omdurman, Ombada, Karary, Bahry and Sharq al Nil*). The sample size was calculated using an estimated impact prevalence of 50%, a design effect of 2, and a precision of 0.06. The minimum sample size to satisfy these requirements was estimated to be 550 children in each school sector with dropouts taken into account (total = 1100). A two stage, stratified (according to gender and locality) cluster sampling design with probability proportional to size and school as the cluster was employed. The cluster size was estimated to 30 students per school. Thirty-seven schools were randomly selected from a total of 1866 schools listed in the area as follows: 8 public boys' schools, 8 public girls' schools, 5 public mixed gender schools, 8 private boys' schools and 8 private girls' schools. All 12-year-olds in the selected schools were eligible for the study. The desired number of children was not always found complete in the randomly selected schools. Extra schools were thus chosen with the criteria of selection being the geographical proximity; 58 schools were eventually visited. A total of 1117 healthy 12-year-old schoolchildren were recruited with the following inclusion criteria; healthy children (attending school on the day of clinical examination and who were free from any serious illness) and those who had not experienced multiple extractions (> 5 missing teeth). Subsequently, to generalise to all 12-year-old schoolchildren in Khartoum state, the whole sample was weighted according to school sector (public/private = 7/1).

### Ethical consideration

Procedures for obtaining consent and ensuring confidentiality were proposed by the ethical research committees in The Sudan. Written permission to conduct the study was thus obtained from the authorities at the Ministry of Health and Ministry of Education, locality administration and individual school administration. Verbal informed consent was obtained from the participants.

### Oral examination

A full mouth oral clinical examination, carried out by a calibrated dentist, was undertaken from October 2007 to February 2008. Calibration exercises for the clinical measures were carried out at the University of Bergen.

Caries was assessed under direct sunlight using the decayed, missing and filled tooth index (DMFT) and in accordance with the WHO caries diagnostic criteria for epidemiological studies. The variable 'active caries' reported later, included decayed teeth diagnosed according to WHO criteria in both deciduous and permanent dentition [16].

The gingival index (GI) [17] and plaque index (PI) [18] were used to assess oral hygiene status. GI was initially coded as follows: 1- normal, 2- mild inflammation, 3- moderate inflammation, 4- severe inflammation. PI was initially coded as follows: 1- no plaque, 2- film of plaque, 3- moderate accumulation, 4- abundant accumulation. GI and PI scores were each categorized into groups: 0 (≤1) and 1 (> 1). The dichotomized variables were then combined such that a score of 1 on both variables was coded as (1) and the other alternative combinations were coded as (0). This meant that children with signs of moderate inflammation (bleeding on probing), and moderate accumulation of plaque on tooth surface, and more were defined as children with poor oral hygiene. Dean's index was used to record dental fluorosis [19]. Cases with no signs of fluorosis were coded (0), and all other signs of fluorosis (questionable, very mild, mild, moderate and severe) were coded as (1). The following were marked as traumatized; teeth with dark discolouration, presence of swelling or fistula adjacent to an otherwise healthy tooth, teeth missing due to trauma and a tooth crown fractured when some of its surface was missing as a result of trauma [16]. Any child with dental trauma was given a score of (1).

### Questionnaire survey and measures

Structured questionnaires were administered by trained field assistants. A pilot study conducted prior to the main study tested the validity of the Adult-OIDP questionnaire. This instrument was designed for 12-year-olds and above, however, the children in this study found the questions complex. Based on these findings, a shift from the adult to the child version of the OIDP was made. Furthermore, the pilot revealed that children were unable to respond appropriately to a self-administered approach, therefore, a shift to a face-to-face interview was made.

#### Child-OIDP

Oral health-related quality of life was measured using an Arabic version of the eight item Child-OIDP questionnaire. The questionnaire, originally constructed in English, was translated into Arabic and back translated by different translators and subsequently the two English versions were compared. They were proclaimed acceptable by the first author. The questionnaire was translated to classical Arabic, but read out to each student individually in a Sudanese dialect to ease the comprehension. Initially, the participating children were first presented with a list of 16 impairments; toothache, sensitive teeth, tooth decay (hole in teeth), exfoliating primary teeth, tooth space (due to a non-erupted permanent tooth), fractured permanent tooth, colour of tooth, shape or size of tooth, position of tooth, bleeding gum, swollen gum, calculus, oral ulcers, bad breath, deformity of mouth or face, erupting permanent tooth and missing permanent tooth. From that list, the schoolchildren selected the impairments they experienced in the past 3 months. Then, they were asked about the frequency and severity of each of the 8 Child-OIDP items, e.g. '*Has your oral health affected your eating habits, speaking, mouth cleaning, relaxing, maintaining your emotional state, smiling, schoolwork and contact with people in the past three months?' *If the schoolchild responded positively, he/she was asked about the frequency and severity of each impact, e.g. "*How often did this happen? How severe was it?' *A single impact frequency scale for individuals affected on a regular basis was used. The frequency and severity of impacts were scored on a 3 point Likert scale (1-3) as follows: Frequency scores (1) being once or twice a month, (2) three or more times a month, or once or twice a week (3) three or more times a week. Severity scores; 1 = little effect, 2 = moderate effect and 3 = severe effect. Lastly, the children were asked to mention the impairments they thought caused the impact on each performance. A maximum of 3 impairments per impact were recorded.

From the frequency scores (range between 1-3) of each of the 8 items, the following variables were constructed as described by Gherunpong et al. [20] and Mtaya et al. [9]:

*Child-OIDP simple count score *(Child-OIDP-SC) or *Extent *(range between 0-8) refers to the number of performances with impacts (PWI) affecting a child's quality of life in the past 3 months. This score was grouped into those with impact (frequency score 1 to 3) and those without impact (score 0).

*Child-OIDP ADD Score *(range between 0-24) is the sum of the reported frequencies (range between 0-3) of the 8 items.

The *Impact Score (range between 0-72) *is the sum of the 8 *Performance Scores (PS) *(range between 0-9). PS is the product of the severity (range between 0-3) and frequency (range between 0-3) scores. The *Overall Impact *is the impact score divided by 72 and multiplied by 100.

Each performance score (range between 0-9) was classified into 6 levels of intensity following the alternative scoring method described by Gherungpong et al [20]; non, very little, little, moderate, severe and very severe impact.

#### Socio-demographics and behavioural factors

The survey included 9 variables on dichotomous indicators of socioeconomic status [12]. Socio-demographics were assessed in terms of parental education and information on household assets. A single variable SES was later calculated using principal component analysis as described elsewhere [14]. SES was assessed by dividing the principal component into quintiles such that each household was classified as lowest, lower, low, middle and higher SES. For the sake of providing a dichotomised variable, the latter two quintiles were combined to predict 'middle' SES and the earlier three for 'low' SES. The questionnaire also contained two global self-rating questions on oral health perceptions; *'What do you think is the state of your mouth and teeth?*' and *'Are you satisfied with the appearance of your teeth?' *with oral health status on 4 points Likert scales ranging from 'very good' and 'good' (interpreted as good) to 'bad' and 'very bad' (interpreted as bad) and 'very satisfied' and 'satisfied' (interpreted as satisfied) to 'not satisfied' and 'not satisfied at all' (interpreted as dissatisfied), respectively. Tooth brushing habits were reported with respect to frequency (everyday once or more, once every 2^nd ^day, once every third day, once a week, irregular or no tooth-brushing at all) and instruments used for brushing (tooth brush, miswak-natural toothbrush made from the twigs of the *Salvadora persica *tree, finger), agents used with brushing (tooth paste, water, other). Dental history was recorded based on history of visit to the dental clinic (have you visited a dental clinic before) and reason for dentist visit (follow-up, pain, other). Sugar-sweetened snack consumption was measured using a food frequency questionnaire on the following seven food items: sweet biscuits, chocolates, popsicles, soft drinks, sticky dessert and sweets. The report was on 3 times a week or more and less than three times. The sum score of all the seven food items was calculated and further dichotomised into 3 items and less, and more than three items. Therefore a child was categorized a high consumer of sugar-sweetened snacks when they consumed more than 3 items, 3 times a week or more.

### Statistical analyses

Statistical analyses were conducted using SPSS 15.0 (SPSS Inc., 2006) and Stata version 10 (StataCorp LP, 2009). Frequencies, means and crude percentage agreement were computed for descriptive purpose. Cohen's Kappa (n = 20) was applied for test-retest reliability and Cronbach's alpha was used for internal consistency reliability. Corrected total and Inter-item correlation were used to assess internal reliability. Multiple variable logistic regression was applied to assess the relationship of the Child-OIDP with socio-behavioural characteristics and clinical oral indicators. Findings reported for all children were weighted according to school sector (public/private = 7/1) to enable generalization to the population of 12-year-olds in Khartoum state. STATA version 10 was used to adjust for cluster sampling, marking the strata as the locality, cluster as the school and the primary sampling unit and the unit of analysis being the schoolchild.

## Results

### Characteristics of participants

Out of the recruited 1117 participants, 1109 responded to the questionnaires (response rate 99%). This sample of 1109 respondents included 50.1% girls (n = 556) and 50.2% public school attendees (n = 556) as opposed to private school attendees. Students' socio-demographic characteristics and clinical parameter scores by school sector are depicted in Table 1.

**Table 1:** Frequency distribution (%) of participants' socio-demographic characteristics dental treatment availability and clinical indicators of private (n = 553) and public (n = 556) school attendees.

Socio-demographic characteristics	Public schools %(n)	Private schools % (n)	P-Value #
**Father's education**	19.9 (111)	4.2 (23)	< .001
Low	52.2 (291)	28.6 (158)	
Medium	26.9 (150)	66.7 (368)	
High			
**Mother's education**	23.3 (130)	3.6 (20)	< .001
Low	62.5 (348)	54.7 (302)	
Medium	13.6 (76)	40.6 (224)	
High			
**Socioeconomic status variable**			
Low	78.8 (434)	49.8 (273)	< .001
Middle	21.2 (118)	50.2 (277)	
**History of dentist visit**	1.1 (6)	3.3 (18)	. <.001
Follow-up\checkup	32.3 (180)	60 (331)	
Pain	66.6 (371)	36.8 (203)	
Never visited			
**Dental treatment experience**			
Extraction only	18.3 (102)	32.6 (180)	< .001
Others	5.6 (31)	11.4 (63)	
**Professional therapy for toothache sought**	18 (100)	38.6 (213)	< .001
**Locality**			
Khartoum	9 (50)	30.4(168)	< .001
Other	91 (506)	69.6 (385)	
**Tooth brushing**			
Regular	89.9(500)	97.3(538)	< .001
Irregular	10.1 (56)	2.7 (15)	
**Sugar-sweetened snack intake**			
High consumer	33.8(188)	32 (177)	< .001
Low consumer	66.2 (368)	68 (376)	
**Past caries experience**			
DMFT > 0	23.6 (131)	30.2(167)	< .001
DMFT = 0	76.4 (425)	69.8 (386)	
**Active caries (permanent and deciduous dentition)**	30.6 (170)	34.7(192)	0.141
Present	69.4 (386)	65.3 (361)	
Not present			
**Fluorosis**			
Present	15.8(88)	8 (44)	< .001
Not present	84.2 (468)	92 (509)	
**Dental trauma**			
present	1.8(10)	2.7 (15)	0.305
Not present	98.2 (546)	97.3 (538)	

# P value for Chi-Square test to compare proportions of socio-demographic characteristics between the two school sectors.

### Psychometric properties of the Child-OIDP

Internal reliability refers to the extent to which a measure is consistent within itself [21]. For the OIDP performance scores, the inter-item correlation coefficients ranged between 0.11 (relationship between smiling and doing school work) and 0.43 (relationship between cleaning teeth and eating) (Table 2). All the coefficients were positive. The standardized Cronbach's alpha coefficient was 0.73 for the whole sample, and 0.78 and 0.67 for public and private school attendees, respectively. The alpha value decreased each time an item was deleted from the model. The corrected item-total correlation values were 0.4 and above for all items.

**Table 2:** Pearson's correlation between single items of the Child-OIDP Performance scores

Performance scores	Eating	Cleaning teeth	Speaking	Smiling	Relaxing	Emotional stability	School work	Social
**Eating**	1							
**Cleaning teeth**	0.43	1						
**Speaking**	0.23	0.21	1					
**Smiling**	0.20	0.17	0.22	1				
**Relaxing**	0.36	0.26	0.21	0.22	1			
**Emotional stability**	0.34	0.28	0.27	0.42	0.30	1		
**School work**	0.20	0.18	0.18	0.11	0.28	0.16	1	
**Social**	0.23	0.22	0.27	0.29	0.22	0.28	0.26	1

All coefficients statistically significant at p < 0.05.

Test-retest reliability refers to the extent of measurement consistency between different points in time. The questionnaire was reintroduced to 20 randomly selected schoolchildren from a single boys' school with a 10-day-interim period. Weighted Cohen's Kappa was 0.70 for eating. The Kappa value was 1.00 for the following Child-OIDP items; speaking, cleaning teeth, relaxing, sleeping, smiling, social contact and emotional state.

All schoolchildren completed Child-OIDP frequency inventory providing support for its face validity. As shown in Table 3, criterion and concurrent validity for the 8 item Child-OIDP inventory was demonstrated, in both public and private school attendees, in that the mean Child-OIDP-SC, Child-OIDP-ADD and overall impact scores increased as children's self reported oral health changed from good to bad and from satisfied to dissatisfied. The results were all statistically significant.

**Table 3:** The Child-OIDP scores by perceived oral health and satisfaction with oral health.

Self-rated oral health measures	Child-OIDP-SC	OIDP-ADD	Overall impact	Independent samples T test
	**Mean **[29]	**Mean **[29]	**Mean **[29]	
**Perceived oral health**				
**Public**				
Good	1.0(1.5)	1.5(2.6)	4.3(8.1)	
Bad	3.1(2.1)	5.2(3.8)	16.7(14.4)	< 0.001
**Private**				
Good	1.1(1.4)	1.8(2.5)	4.9(7.8)	
Bad	2.6(1.8)	4.6(3.5)	14.8(13.0)	< 0.001
**Satisfaction with oral health**				
**Public**				
Satisfied	1.0(1.6)	1.6(2.8)	4.5(9.0)	
Not satisfied	2.8(2.0)	4.6(3.5)	14.3(13.3)	< 0.001
**Private**				
Satisfied	1.1(1.4)	1.8(2.6)	4.8(8.1)	
Not satisfied	2.3(1.8)	4.0(3.3)	12.8(12.3)	< 0.001

### Prevalence, extent and intensity of oral impacts

The weighted prevalence estimate of the Child-OIDP amounted to 54.6%. The corresponding (not weighted) estimates in private and public school attendees were 64% and 53.4%. A total of 18.1% reported one impact, 11.7% reported two impacts, 10.5% reported three impacts, 6.4% reported four and the remaining 7.9% reported more than four impacts. With respect to sector, the private versus the public school attendees' report for 1,2,3,4 and more impacts was as follows: 23.6% vs 17.5%,16.3% vs 11.0%, 11.4% vs 10.5%, 6.2% vs 5.5% and 6.4% vs 78.%, respectively.

In the weighted sample, the highest impact was reported on eating (35.5%) followed by cleaning (28.3%) and the lowest impacts were on speaking (8.6%) and social contact (8.7%) (Table 4). Private school attendees reported the highest and lowest impacts on eating (40%) and speaking (4.3%), respectively. Public school attendees reported highest impact on eating (34%) and the lowest impact on both social contact and speaking (9.2%). Reported impacts on smiling and emotional status differed statistically significantly between public and private school attendees (p < 0.05). There were no significant differences between girls and boys in any performance. The intensity of impact is illustrated in Table 5 for the total study group. Most private (44.1%) and public (46.4%) school attendees' reports on impact were of moderate intensity.

**Table 4:** OIDP prevalence, Performance score and Child-OIDP mean for the 8 items on the Child-OIDP scale (n = 1109).

	Overall	Eating	Speaking	Cleaning	School	Smiling	Emotion	Relax	Contact
	n = 1109	n = 415	n = 75	n = 312	n = 85	n = 214	n = 265	n = 192	n = 80
**OIDP prevalence %(all)**	54.6	35.6	8.6	28.3	8.9	16.0	20.3	17.7	8.7
**Performance** **score** **Range**	0-9	0 - 9	0 - 9	0 - 9	0 - 9	0 - 9	0 - 9	0 - 9	0 - 9
**Mean **[29]	1.5 (2)	1.3 (2)	0.3(1)	1.0 (2.0)	0.3(1.0)	0.7 (1.9)	0.7 (1.8)	0.6(1.6)	0.3 (1.2)
	**Overall**	**Eating**	**Speaking**	**Cleaning**	**School**	**Smiling**	**Emotion**	**Relax**	**Contact**
	**n = 556**	**n = 194**	**n = 51**	**n = 158**	**n = 52**	**n = 89**	**n = 107**	**n = 99**	**n = 51**
**OIDP prevalence %** **(Public school attendees)**	53.4*	35.0	9.2	28.4	9.4	16.0*	19.2 *	17.8	9.2
	**Overall**	**Eating**	**Speaking**	**Cleaning**	**School**	**Smiling**	**Emotion**	**Relax**	**Contact**
	**n = 552**	**n = 221**	**n = 24**	**n = 154**	**n = 33**	**n = 125**	**n = 158**	**n = 93**	**n = 29**
**OIDP prevalence % (Private school attendees)**	64.0	40.0	4.3	27.8	6.0	22.6	28.6	16.8	5.2

* Chi square P < 0.05

**Table 5:** Percentage of Impact intensity for the 8 items on the Child-OIDP scale for private and public school attendees (n = 1109).

Impact intensity (%)	Eating n = 415	Speaking n = 75	Cleaning n = 312	School n = 85	Smiling* n = 214	Emotion* n = 265	Relax n = 192	Contact* n = 80	Total %
**Very little**									
Private	5.1	0.7	5.6	1.3	2.0	5.1	3.1	1.4	24.3
Public	6.7	1.6	7.0	2.2	2.0	2.7	3.2	1.8	27.2
**Little**									
Private	13.9	1.3	8.1	2.0	4.3	8.3	2.4	1.4	41.7
Public	9.5	2.2	7.6	1.3	4.3	4.5	3.6	1.8	34.8
**Moderate**									
Private	9.8	1.4	8.5	2.2	5.8	7.4	8.1	0.9	44.1
Public	9.7	3.6	7.2	4.5	4.0	6.8	6.8	3.8	46.4
**Severe**									
Private	7.4	0.5	4.0	0.4	4.7	4.9	2.2	0.5	24.6
Public	5.4	0.9	3.4	1.3	3.2	3.6	2.9	0.9	21.6
**Very severe**									
Private	3.8	0.4	1.4	0.2	5.6	2.9	1.1	0.9	16.3
Public	3.6	0.9	3.1	0.2	2.5	1.6	1.1	0.9	13.9

* Difference between reports from different school sectors is statistically significant. Chi square P < 0.05

### Causes of oral impacts

**Figure 1:**
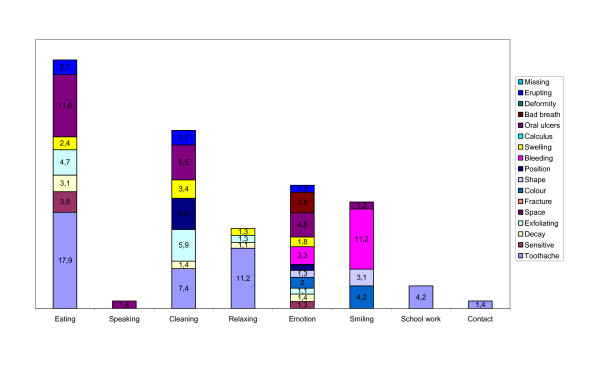
**Percentage contribution of perceived impairments associated with performances in public school attendees**. (contributions of less than 1% were excluded).

**Figure 2:**
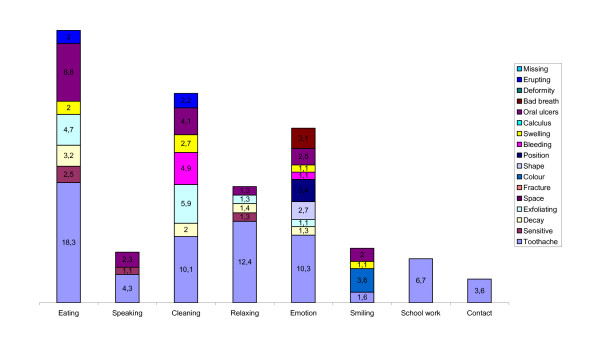
**Percentage contribution of perceived impairments associated with performances in private school attendees**. (contributions of less than 1% were excluded).

The impairments perceived to cause the impacts on each of the 8 performances are shown for public and private school attendees in Figures 1 and 2. The most commonly reported impairment was erupting teeth followed by toothache. The impairments that contributed to all the 8 impacts were toothache, sensitive teeth, exfoliating teeth, swollen gums and bad breath. The most commonly reported impact was on eating and the most commonly associated impairment with this was toothache followed by oral ulceration. Toothache was the most frequently associated cause of almost all impacts in both private and public school attendees. In private school attendees, the majority of impacts on smiling were attributed to colour while for public school attendees, bleeding was the main cause. Among all children, colour was the most frequently reported cause of impact on emotional status.

The Child-OIDP-SC was regressed on socio-demographics, behavioural and clinical oral health indicators using bivariate and multiple variable logistic regression analyses (Table 6).

**Table 6:** Child-OIDP (0 = no impacts, 1 = at least one impact) regressed on socio-demographics, behavioral- and clinical oral health indicators: odds ratio (OR) and 95% Confidence interval (CI), unadjusted and adjusted analyses.

	Unadjusted	Adjusted Nagelkerke R^2 ^= 0.350 Public school attendees n = 514	Adjusted Nagelkerke R^2 ^= 0.175 Private school attendees n = 531	Adjusted Nagelkerke R^2 ^= 0.254 Whole sample n = 1045
***Socio-demographic data***				
**Gender**				
Boy	1	1	1	1
Girl	0.8(0.7-1.1)	0.9 (0.6-1.3)	0.9 (0.6-1.3)	0.8 (0.6-1.1)
**School sector**				
Public	1			1
Private	1.6(1.2-2.0)*			1.2(0.9-1.7)
**Locality**				
Other	1	1	1	1
Khartoum	1.3(1.0-1.8)*	1.5(0.7-3.0)	1.1(0.7-1.7)	1.2(0.8-1.7)
**SES**				
Low	1	1	1	1
Middle	1.4(1.1-1.8)*	1.9(1.1-3.1)*	1.0(0.7-1.5)	1.3(0.9-1.7)
***Behavioral variables***				
**Tooth-brushing frequency**				
Irregular	1.0(0.6-1.7)			
Daily				
**History of dentist visit**	1	1	1	1
No	0.6(0.4-0.7)*	0.9(0.5-1.4)	0.9(0.6-1.3)	0.8(0.6-1.1)
Yes				
**Satisfaction with oral health**				
Not satisfied	1	1	1	1
Satisfied	0.2(0.1-0.2)*	0.2(0.1-0.5)*	0.6(0.3-0.9)*	0.4(0.3-0.6)*
**Perception of oral health**				
Bad	1	1	1	1
Good	0.1(0.1-0.2)*	0.2(0.1-0.5)*	0.3(0.2-0.5)*	0.3(0.2-0.4)*
**Sugar-sweetened snack intake**				
≤3 items/week	1	1	1	1
> 3 items/week	1.6 (1.2-2.0)*	1.4 (0.9-2.1)	1.4 (0.9-2.2)	1.4 (0.9-1.8)
***Clinical parameters***				
**Mean GI index**				
Score ≤ 1	1	1	1	1
Score > 1	1.3(1.0-1.7)*	1.2 (0.7-1.9)	1.5 (0.9-2.5)	1.3 (0.9-1.8)
**Mean PI index**				
Score ≤ 1	1	1	1	1
Score > 1	1.3(1.0-1.7)*	1.1(0.6-2.0)	1.3(0.8-2.1)	1.3(0.9-1.8)
**Dean's Index**				
Score = 0	1			
Score > 0	1.1(0.7-1.5)			
**Caries experience**				
DMFT = 0	1	1	1	1
DMFT > 1	1.5(1.1-1.9)*	0.9(0.6-1.5)	1.4(0.9-2.2)	1.2(0.9-1.6)
**Active caries**				
No	1	1	1	1
Yes	2.5(1.9-3.4)*	3.5(2.1-5.6)*	1.2(0.7-1.8)	2.0(1.4-2.6)*
**Dental trauma**				
No	1			
Yes	1.5(0.6-3.5)			

* Chi square P < 0.05

All variables that showed statistically significant association with OIDP in unadjusted analysis; SES, satisfaction with oral health, perception of oral health, frequency of sweetened snack intake, mean GI, mean PI, caries experience and active caries were inserted into the multiple variable logistic regression analysis model.

The variables gender, tooth-brushing frequency, fluorosis and dental trauma did not show significant association in unadjusted analyses. However, gender was reinserted in the multiple variable logistic model for its importance as a socio-demographic variable, in addition to it maintaining a statistical p-value of less than 0.2 [22]. The model based on the total sample explained 25% of the variance (Nagelkerke R^2 ^= 0.254) when all the selected variables were inserted simultaneously. The model explained 35% of the variance for public school attendees, and 18% for private school attendees.

After adjusting for confounders, satisfaction with and perception of oral health maintained significance in all three models; thus providing further support to the validity of the instrument. Active caries maintained a significant association with the whole sample (OR 2.0 95% CI 1.4-2.6) and public school attendees (OR 3.5 95% CI 2.1-5.6).

SES was associated with public school attendees Child-OIDP only (OR 1.9 95% 1.1-3.1).

## Discussion

This report provides new and detailed evidence of the Child-OIDP of public and private school attendees in Khartoum state, Sudan. An Arabic version of the CPQ _11-14 _has been validated in 11 to 14-year-olds in Saudi Arabia [23]. However, Brown et al. (21), acknowledged the limitations of the Arabic CPQ in that it was lengthy and included some questions that were not pertaining to the Saudi and Sudanese children such as the difficulties associated with playing musical instruments. Thus, it was preferred to translate the Child-OIDP to the Arabic language. This study presents the first attempt to evaluate the psychometric properties of an Arabic version of the Child-OIDP and is the second report on children's OHRQoL from an African context [9]. The psychometric properties of OHRQoL inventories depend largely on the linguistic and cultural attributes of the population under study. A need for testing each instrument when applied in a new socio-cultural context has been acknowledged [24].

Public and private school attendees differed significantly in their socio-behavioural and clinical characteristics (Table 1). Moreover, private school attendees were the minority in the population (12%) and their schools tended to be geographically centrally located and better equipped with respect to school materials when compared to their public school counterparts in the same locality. For these reasons, analyses were stratified by school sector.

When applied to 12-year-old Sudanese schoolchildren attending private as well as public primary schools, the Child-OIDP showed acceptable psychometric properties and is considered a valid, reliable and practical inventory for use in this population. The standard alpha coefficient was above the recommended threshold of 0.7 [21]. Corresponding figures from Thailand, Tanzania, Spain, France and England regarding Cronbach's alpha were 0.82, 0.77, 0.68, 0.57 and 0.58, respectively. The correlation coefficients were all positive and above or equal to the recommended level of 0.2, with the exception of the correlation between smiling and each of school work (0.11) and cleaning (0.17) [25]. Test-retest reliability was confirmed as the weighted kappa indicated very good reliability for all performances. The present results provided support for the concurrent validity of this instrument. The Child-OIDP was constructed upon a solid theoretical basis and the content validity has been further sufficiently evaluated in other populations [6,8,10].

Active caries was associated with reported oral impacts (Child-OIDP score > 0) in unadjusted and adjusted logistic regression analysis in the total sample and in public school attendees (P < 0.05) (Table 6). Pain, discomfort, functional and aesthetic limitations are known to usually accompany active caries, providing explanation to our findings. This variable was constructed to focus on decay, a component which is diluted in a measure of past caries experience like the DMFT, because of the inclusion of restored and missing teeth components in it. Furthermore, DMFT measures the experience in permanent teeth only while in this study the variable 'active caries' included lesions in deciduous teeth as well. Other studies have reported associations between past caries experience, in the form of DMFT, and OHRQoL [26,27]. These findings further stress the necessity for provision of dental care in the population investigated.

A higher SES status in this study reflected a higher level of education, a higher social status in terms of parental occupation and better living standards in terms of better household conditions and properties. As opposed to the situation pertaining to the total sample and private school attendees, public school attendees with middle level SES were almost twice as likely to report oral impact on daily performance compared to their counterparts with low SES independent of oral diseases (Table 6). A study of Canadian children reported SES disparities in OHRQoL, where children of a lower SES reported the higher impact [28]. Thus, it may be deduced from our study that the understanding of the public school attendees' need for good OHRQoL increases with an increase in their SES. This might also reflect higher expectation with respect to having a good dentition status among affluent compared to non-affluent 12-year-olds in Khartoum. Their better knowledge and awareness of better opportunities for oral health care may account for their report on the high impact, and thus reflects their demand for a better OHRQoL.

A Medline search was conducted with the following terms C-OIDP, Child-OIDP and child oral impacts on daily performance, to find all published studies that have applied the Child-OIDP instrument. Table 7 illustrates a brief comparison. The prevalence of oral impacts on daily performance in the Sudan (54.6%) was almost twice as much compared to that reported in a similar age group in Tanzania (28.6%). With the exception of the UK, all the remaining countries had higher impact prevalence, emphasizing the socio-cultural variation in the Child-OIDP. Despite the high prevalence of impact on daily performance compared to Tanzania, the intensity of the impact was rarely high among Sudanese schoolchildren where most reports had a magnitude of little or moderate intensity, and private school attendees reported a higher frequency of higher intensities (severe and very severe) compared to their counterparts.

**Table 7:** A comparison between published Child-OIDP reports. Child-OIDP mean is the mean of the OIDP sumscore.

	Year	Mean age	Mean Child-OIDP score	Impact > 0 (%)	Performances with highest impact	Most common reported causes
**Thailand**	2009	12	7.8(7.8)	85.2	Eating Emotional stability	Sensitive tooth Oral ulcer Toothache
**France**	2005	10	6.3(8.2)	73.2	Eating Speaking	Badly positioned tooth Oral ulcer Erupting tooth Bleeding gums
**UK**	2006	10-11	NR	40.4	Eating Cleaning	NR
**Tanzania**	2007	13	NR	28.6	Eating Cleaning	Toothache Ulcer in mouth Position of teeth
**Peru**	2008	11-12	NR	82.0	Eating Cleaning	Toothache Sensitive teeth Bleeding gums
**Brazil**	2008	11-14	9.2(10.1)	80.7	Eating Emotional status	Sensitive teeth Tooth colour
**Italy**	2009	11-16	1.9(3.7)	94.5	Eating Cleaning	Sensitive teeth Tooth ache Tooth decay
**Spain**	2009	11-12	2.7(5.6)	36.5	Eating Cleaning teeth	Sensitive teeth Toothache
**Sudan**	current study	12	1.4(1.7)	54.6	Eating Cleaning	Erupting teeth Tooth ache

NR: Not reported

The difficulty with eating was the most important aspect of Sudanese schoolchildren's Child-OIDP. This is in accordance with results reported in other studies [7-9,11-13,29,30]. Moreover, in contrast to other reports, Sudanese children reported erupting teeth (39.6%) as the most frequent cause of oral impacts. However, this impairment may be overlooked since it is a natural process that cannot be avoided at this age and will subside eventually, and so the next most reported impairment was toothache (38.5%). The high report on toothache, bleeding gums and oral ulcers reflects upon their knowledge of oral and functional problems and indirectly on their information of treatment availability.

Children's concern about their aesthetic appearance becomes significant when they approach adolescence [31]. Contrary to this, our study suggests that oral appearance was not one of the main concerns of this population because the two least reported impacts were on the social performances, social contact and smiling and the least reported impairments were deformity, fracture, missing, space, shape, position and colour. The cultural norms and expectations influence the perception of oral health and its effect on their quality of life. The schoolchildren could be unfamiliar with opportunities for improvement of appearance as a result of lack of oral health education and shortage in accessible dental health services.

A limitation of this study is in its cross-sectional design, making it difficult to draw any conclusion about causes and effects. Further longitudinal studies are needed to better understand and interpret OHRQoL measures in children; although these are difficult to conduct in developing countries due to financial restraints and lack of population records.

In conclusion, the Arabic Child-OIDP showed acceptable psychometric properties and is considered a valid, reliable and practical inventory for use in this population. Almost half of the population reported an impact on their quality of life, mostly on the eating performance with the most associated impairments being erupting teeth and toothache. Child-OIDP was not only determined by oral disease in the whole population, but also by the socio-behavioural variables SES in public school attendees. Despite the low prevalence of the dental caries pathology (24%), a significant relationship with an average moderate intensity was found with Child-OIDP. Oral appearance was not one of the main concerns of this population.

A comprehensive assessment of oral health is useful to oral healthcare policy makers for vital planning of oral healthcare programmes in order to promote health resources and address oral health needs and demands. Focus in this population should be on oral health education, improving knowledge of the prospective treatment opportunities and provision of such services.

## Competing interests

The authors declare that they have no competing interests.

## Authors' contributions

NNM designed the study and carried out the data collection, data analysis and writing of the article. AAN, TTA and AMF supervised and assisted in writing/editing of the article. All authors have read and approved the final manuscript.
